# Using parallel pre-trained types of DCNN model to predict breast cancer with color normalization

**DOI:** 10.1186/s13104-021-05902-3

**Published:** 2022-01-10

**Authors:** William Al Noumah, Assef Jafar, Kadan Al Joumaa

**Affiliations:** Department of Informatics, Higher Institute for Applied Sciences and Technology, Damascus, Syria

**Keywords:** Breast cancer, Medical image analysis, Deep learning application, Histopathological images, Deep convolutional neural networks, Transfer learning, Image classification, Vahadane, Label smoothing

## Abstract

**Objective:**

Breast cancer is the most common among women, and it causes many deaths every year. Early diagnosis increases the chance of cure through treatment. The traditional manual diagnosis requires effort and time from pathological experts, as it needs a joint experience of a number of pathologists. Diagnostic mistakes can lead to catastrophic results and endanger the lives of patients. The presence of an expert system that is able to specify whether the examined tissue is healthy or not, thus improves the quality of diagnosis and saves the time of experts. In this paper, a model capable of classifying breast cancer anatomy by making use of a pre-trained DCNN has been proposed. To build this model, first of all the image should be color stained by using Vahadane algorithm, then the model which combines three pre-trained DCNN (Xception, NASNet and Inceptoin_Resnet_V2) should be built in parallel, then the three branches should be aggregated to take advantage of each other. The suggested model was tested under different values of threshold ratios and also compared with other models.

**Results:**

The proposed model on the BreaKHis dataset achieved 98% accuracy, which is better than the accuracy of other models used in this field.

**Supplementary Information:**

The online version contains supplementary material available at 10.1186/s13104-021-05902-3.

## Introduction

Breast cancer occur because of abnormal cell division, which develops amass called a tumor [1, 2]. There are two types of tumors: cancerous (malignant) and noncancerous (benign). The detection of breast abnormalities begins firstly with screening methods by taking images of the breast using different types of medical imaging [3, 4]. Secondly, in case that a tumor is found in the images, the type of this tumor must be examined to see if it is benign or malignant, by taking a biopsy from the tumor to be studied by a pathologist [5–7]. For routine diagnosis, pathologists prefer the use of Hematoxylin and Eosin (H&E) [8, 9] to view details of cellular and tissue structure. This routine is considered stressful and hard. It requires a long time and a lot of effort, beside to that classifying the type of tumor depends on the experience of the pathologist. Due to this fact, researchers and doctors have recently begun to benefit from computer-assisted interventions with the help of machine learning techniques [10]. Deep learning algorithms with deeper layers such as DCNN have recently shown success in various medical image analysis tasks such as breast cancer detection and classification [11, 12]. In this paper, a deep learning model will be presented using pre-trained deep neural networks then fine-tuning these networks to be able to recognize histopathological images of breast tumors and determine the nature of the tumor, whether it is benign or malignant.

## Main text

### Materials and methods

#### Dataset

A well-known database called BreaKHis has been used in this paper [13]. This database contains microscopic biopsy images of benign and malignant breast tumors. These images were generated from breast tissue biopsy slides stained with Hematoxylin and Eosin (H&E). The number of images in the database is 7909 images for about 82 patients. Each image is colored within 3 RGB channels of size with dimension (460 pixels height and 700 pixels width) using magnification factors 40, 100, 200 and 400. The dataset currently contains four histological distinct types of benign breast tumors: (A), (F), (PT), and (TA) and the total number of benign images is 2480, and four malignant tumors (breast cancer): (DC), (LC), (MC) and (PC) and the total number of images is 5429. To reduce the number of classes to only two classes, the benign images have been grouped into one class and the malignant images have been grouped into another class.

#### Data preparation

In this phase, the size of images is resized to (244 244), due to speeding up the learning process. Where the original size of images needs a server with high resources. Since the images being processed are images of cancerous tissue, they are not affected by image transforming, inverting, zooming in, or rotating by 90. So, the training data in this phase were augmented using the image generation method. This image data augmentation is a technique used to artificially expand the size of the training data set by creating modified versions of the data set images in order to improve the performance and ability to generalize the model. The transformed operations used are Random Zoom Augmentation with value 2, Random Rotation Augmentation with a value of 90° and Horizontal and Vertical Flip Augmentation.

#### Stain normalization

The first pre-processing step of the proposed pipeline is stain color normalization. A stain normalization method allows transforming image into another image by removing all intensity values from the image while preserving color values. The color response of digital scanners, material and manufacturing technology of the staining supplier, and different staining protocols in the different labs, may cause large color differences in the histopathological images. Therefore, stain normalization is a fundamental and necessary step in the pre-processing of H&E stained breast cancer histopathology images. To solve the stain variability of BreaKHis dataset, the stain normalization pre-processing of histopathological images is firstly carried out using Vahadane method [14]. Vahadane proposed a solution for both stain separation and color normalization. This solution preserves biological structure information by modelling stain density maps based on the following properties: Non-negativity, Sparsity and Soft-classification.

#### Model architecture

**Fig. 1:**
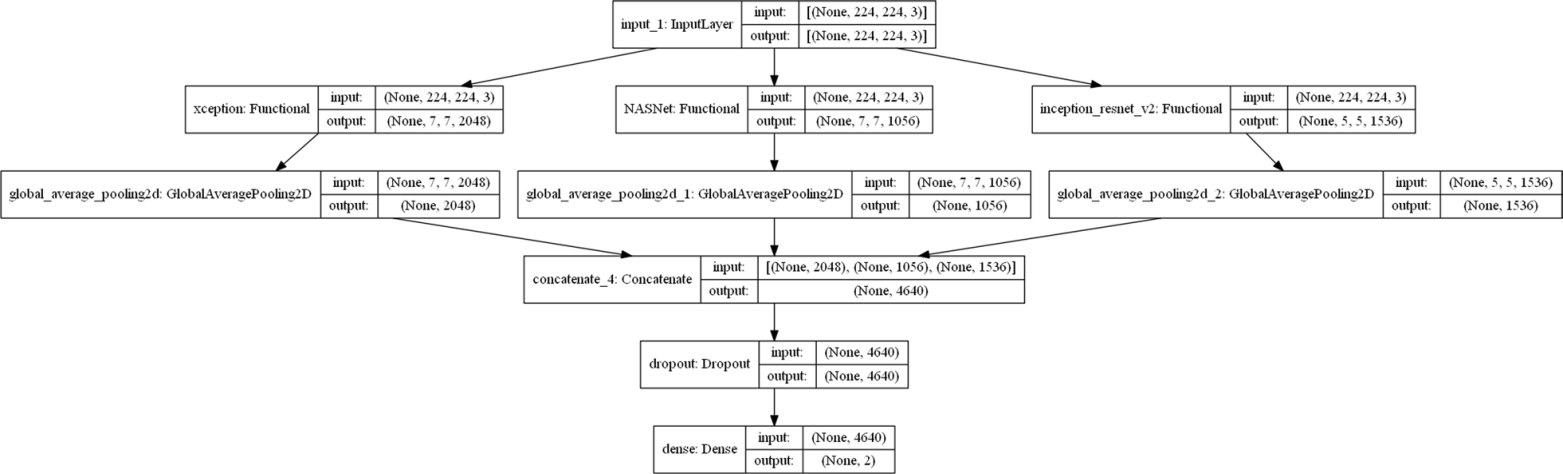
The proposed model architecture and the output vector for each layer represent the outputs for each of the pre-trained models

The model architecture is displayed in Fig.  1, and can be explained as follow:

- The suggested model consists of three pre-trained deep convolutional neural networks (DCNN) which work in parallel as (xception, NASNet and inceptoin_resnet_V2).
- The output of each branch is passed on to a global average pooling layer.
- The output of these three layers are concatenated into a single layer with an output of 4640 neurons, which is an input of a dropout layer with a rate of 0.1.
- Dense layer is used to convert the 4640 neurons to only two-classes benign and malignant.

The outline of these four steps is to concatenate the outputs of each pre-trained model to a stacked ensemble model with output of a three-dimensional vector connected to a dense layer activated by a SoftMax function.

Configures the model for training using these arguments: Adam optimizer with (lr = 0.0001 and decay = 0.00001), label soothing with rate 0.1.

So, the proposed model creates an ensemble model gathering the knowledge of many DCNN classifiers in a single model. Variance and bias are also reduced, thus minimizing the number of errors.

### Results

To train and test the model, the used dataset will be randomly separated into 80% training (1984 Benign images from 2480 and 4343 Malignant images from 5429) and 20% testing (496 Benign images from 2480 and 1086 Malignant images from 5429).

#### Training the model

The suggested model was trained on the data set which is mentioned above, to tune the hyperparameters, a batch size value of 32 is used, also some data generation functions are applied to allow the network to “see” more diversified, but still representative data points during training. Then 20% of the training set will be separated to validate the suggested model as a validation set. The training process should be executed for many epochs. In this paper the validation accuracy achieved a value 97.9% when the model was trained for 45 epochs, and the loss function was 0.5. Within each epoch, the data is fed by batches, when all the batches are fed and training is executed, the epoch is completed. Neural networks are fed by batches for training and testing, preprocessing and feeding them to the neural networks with a batch size. As a single output node for binary classification activates a SoftMax function, the loss function is binary cross-entropy using label smoothing. In each training epoch, the parameters of the model are adjusted in order to obtain better training accuracy compared to the validation set. And so, the epochs continue to be repeated until all epochs of training are completed.

A learning curve is a plot of model learning performance over experience and time. The model can be evaluated on the training dataset and on a holdout validation dataset after each update during training, and plots of the measured performance can be created to show learning curves. Generally, a learning curve is a plot that shows time on the x-axis, and improvement on the y-axis. The validation learning curve calculated from a hold-out validation dataset that gives an idea of how well the model is generalizing. The train learning curve and the validation learning curve for 45 epochs. And both values of the training loss and the validation loss decreased to a point of stability, with a gap between them, this gap is referred to as the generalization gap. All the mentioned curves can be checked in Additional file 1.

#### Testing model

The model was tested in two ways, with and without augmentation. The used augmentation functions are mentioned in “Data preparation”, and the parameters adopted in the test are Precision, Recall, and F1-Score. The testing process was under the condition that, to predict a benign result, the percentage of this prediction should be over 60%, otherwise it is malignant. The results of testing were: The overall accuracy of the suggested model is 98%, the weighted average of precision is 98% and the weighted average of Recall is 98% that with and without augmentation. In Additional file 1 shows the tables of results and the confusion matrices of the test with and without augmentation, respectively.

And in Additional file 2 contain some images classified as true-positive or true-negative (these images are classified correctly). and some of images classified as false-positive or false-negative (misclassified) and probability for each class.

**Fig. 2:**
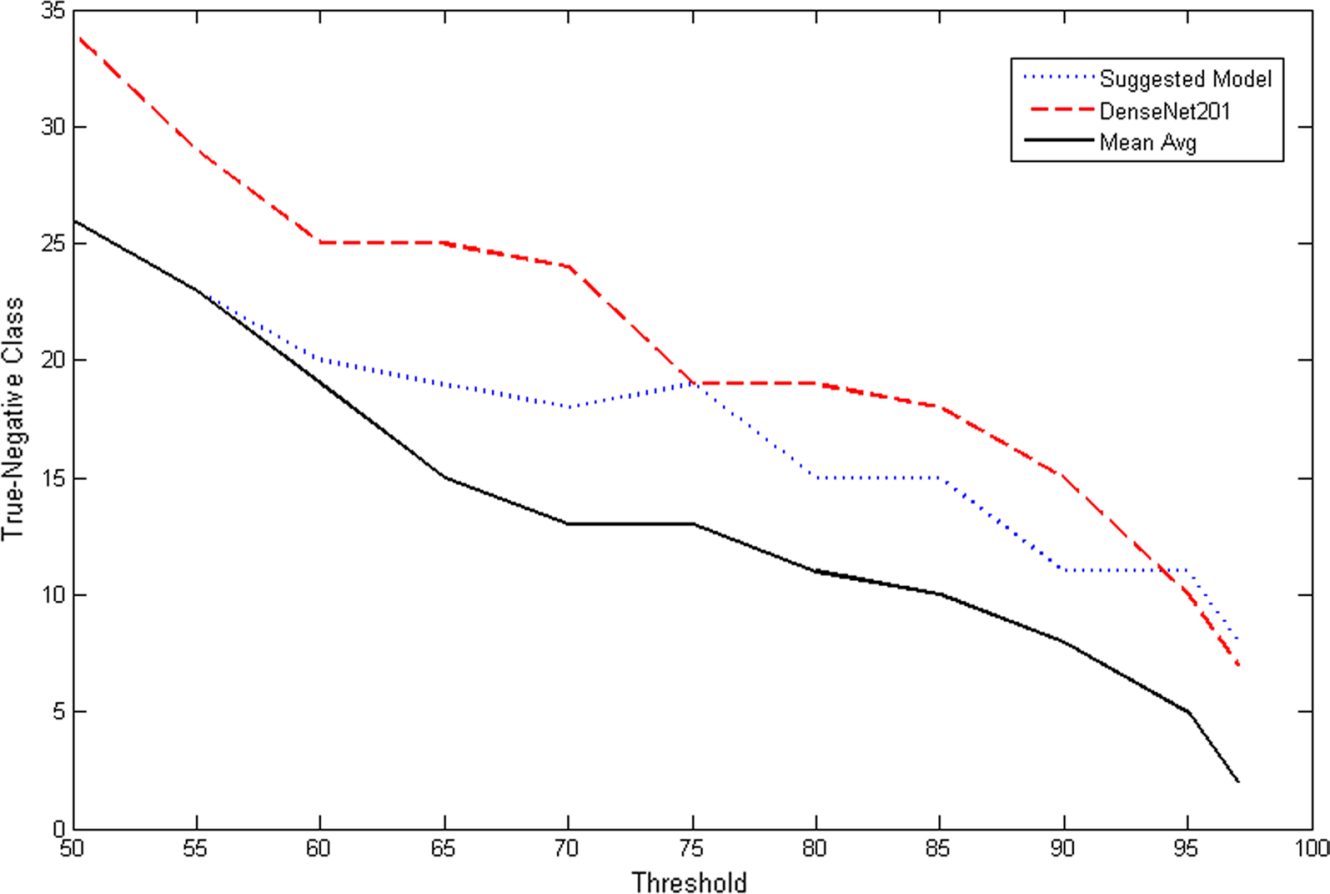
Curves of benign classification changes with threshold

**Table 1:** Changing the statistical values of the suggested model (SM) in comparison with the Densenet model (DM) and the mean average of both models together (AM)

Threshold (%)	Mis class benign			Mis class malignant			Mis class			Accuracy		
	SM	DM	AM	SM	DM	AM	SM	DM	AM	SM (%)	DM (%)	AM (%)
50	26	34	26	14	19	15	40	53	41	97	97	97
55	23	29	23	14	21	17	37	50	40	98	97	97
60	20	25	19	14	21	18	34	46	37	98	97	98
65	19	25	15	16	22	23	35	47	38	98	97	98
70	18	24	13	18	24	24	36	48	37	98	97	98
75	19	19	13	17	27	26	36	46	39	98	97	98
80	**15**	19	11	**23**	29	32	38	48	43	**98**	97	97
85	15	18	10	30	34	39	45	52	49	97	97	97
90	11	15	**8**	33	39	**41**	44	54	49	97	97	**97**
95	11	10	5	54	49	55	65	59	60	96	96	96
97	8	7	2	57	60	65	65	67	67	96	96	96

Bold number indicate best result

#### Improving the model due to the threshold ratio

Traditional classification algorithms often don’t consider the factor of misclassification cost, which leads to classification results that tend to focus on the learning of uncritical class (False-Positive) while ignoring the learning of critical class (False-Negative). But when dealing with issues related to medical diagnosis, the misclassification of one class must have a different cost than the other, because the result of this classification may be related to the patient’s life. When the tumor is diagnosed as benign, this will end the analysis process, while it diagnosed as malignant will allow the pathologist to ensure the validity of this decision using his experiences. The dangerous issue is when the tumor is malignant while the decision of classification model is benign. So that the cost of misclassification for benign cases should be focused more. In this paper, to show the importance of the misclassification of benign class, the threshold ratio of the prediction of the benign decision will be taken into consideration. This step will be implemented in three ways:

1. The suggested model will be tested for a threshold ratio ranging from 50 to 97%.
2. DenseNet201 model [15] will be trained and tested for both, the same training and testing set, and the same range of threshold ratio.
3. The mean average of DenseNet201 output vector, and the suggested model output vector will be tested for the range of threshold ratio mentioned above.

Figure 2 and Table 1 shows the ranging of the threshold ratio for the three ways above.

The results show that the misclassification of the benign cases for different threshold ratios in the suggested model and the mean average were less than DenseNet201 model, in spite of the DenseNet201 model is more complex and more commonly used for this type of problem. So as a result, the suggested model combines simplicity in design and reliability in the decision.

### Conclusions

In this paper, a model that is able to learn using three pre-trained deep neural networks, and concatenate the last layer of these networks with applying label smoothing is presented. Then this model is fine-tuned to be able to recognize H&S pathological images of breast tumors and determine the nature of the tumor, whether it is benign or malignant. The proposed model structure is characterized by the creation of a new structure that is able to take advantage of the power of many pre-trained models and transfer learning from them. This model will be more compatible with histopathological images by applying the application Vahadane algorithm for stain normalization to avoid the problems that appear due to the color disparity between images. The obtained model can distinguish between features of nuclei and tissue features so that the model can be generalized to other types of tissue images. the suggested model achieved an accuracy of 98%, which is better than the results of other models used in this field as DensNet201. the simplicity and accuracy of the suggested model could be considered as a kernel of a system capable of supporting pathologists’ decisions.

## Limitations

The suggested model can aid effectively in the tedious task of determining the pathology, but it is necessary to note that the final diagnosis should be confirmed by the pathologist, due to the importance of the patient’s health and life.

## Supplementary Information

**Additional file 1.** Tables and figures resulted from training and testing the suggested model on the dataset with three section: the first section contains a table for image distribution by magnification factor and class, the second section contains the training and validation curves, and the third section contains the test results with and without augmentation.

**Additional file 2.** Images which are classified correctly and some images which are classified incorrectly with the probability of each image.

## Data Availability

The BreaKHis dataset that supports the findings of this study is available from https://web.inf.ufpr.br/vri/databases/breast-cancer-histopathological-database-breakhis

## Abbreviations

DCNN: Deep convolutional neural network
CT: Computed tomography
MRI: Magnetic resonance imaging
PET: Positron emission tomography
DM: Digital mammography
ML: Machine learning
DL: Deep learning
H&E: Hematoxylin and Eosin
CAD: A computer-aided diagnosis
A: Adenosis
F: Fibroadenoma
PT: Phyllodes tumor
TA: Tubular adenona
DC: Ductal carcinoma
LC: Lobular carcinoma
MC: Mucinous carcinoma
PC: Papillary carcinoma
SM: The suggested model
DM: The Densenet model
AM: The average model
