# Supplementary material for: Using parallel pre-trained types of DCNN model to predict breast cancer with color normalization
Source: BMC Res Notes. 2022 Jan 10;15:14. doi: 10.1186/s13104-021-05902-3 (PMC8751220; doi:10.1186/s13104-021-05902-3)
Supplement: Supplementary file 1 — Additional file 1. Tables and figures resulted from training and testing the suggested model on the dataset with three section: the first section contains a table for image distribution by magnification factor and class, the second section contains the training and validation curves, and the third section contains the test results with and without augmentation. [file 13104_2021_5902_MOESM1_ESM.pdf]

Section 1: Images were taken for about 82 patients with different magnification factors as shown in table1:

Table 1: Image distribution by magnification factor and class

Magnification	Benign	Malignant	Total
40×	625	1370	1995
100 ×	644	1437	2081
200 ×	623	1390	2013
400 ×	588	1232	1820
**Total**	**2480**	**5429**	**7909**
Patients	24	58	82

Section 2: The train learning curve and the validation learning curve for 45 epochs are shown in Figure 1 (a) and the training loss and validation loss curve are shows in Figure 1 (b).

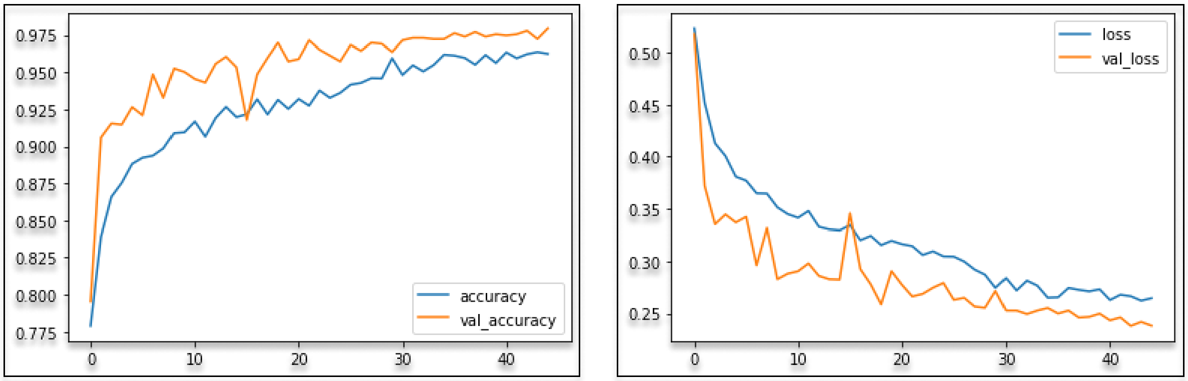

(a) The validation and training accuracy function

(b) The validation and training loss function

Figure 1 The training and validation curves

Section 3: Table 2 shows the results of the test without augmentation, while table 3 shows the result with augmentation. The confusion matrix for breast cancer without and with augmentation method are shown in Figures 2 (a) and (b) respectively.

Table 2 Test Result with augmentation

	**Precision**	**Recall**	**F1-Score**	**Support**
Benign	0.97	0.96	0.97	496
Malignant	0.98	0.99	0.98	1086
**Accuracy**			**0.98**	1582
Macro AVG	0.98	0.97	0.97	1582
Weighted AVG	0.98	0.98	0.98	1582

Table 3 Test Result without augmentation

	**Precision**	**Recall**	**F1-Score**	**Support**
Benign	0.97	0.95	0.96	496
Malignant	0.98	0.99	0.98	1086
**Accuracy**			**0.98**	1582
Macro AVG	0.97	0.97	0.97	1582
Weighted AVG	0.98	0.98	0.98	1582

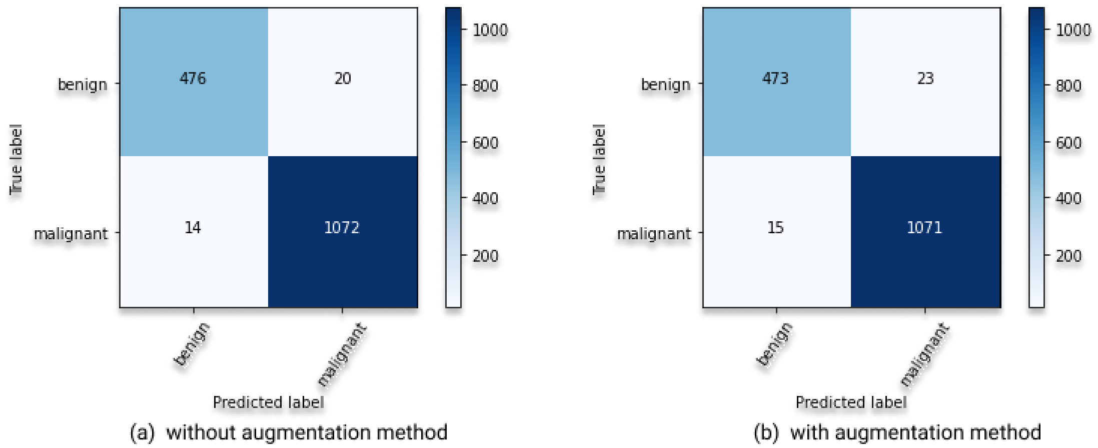

Figure 2 Confusion matrices
