# Supplementary material for: Using parallel pre-trained types of DCNN model to predict breast cancer with color normalization
Source: BMC Res Notes. 2022 Jan 10;15:14. doi: 10.1186/s13104-021-05902-3 (PMC8751220; doi:10.1186/s13104-021-05902-3)
Supplement: Supplementary file 2 — Additional file 2. Images which are classified correctly and some images which are classified incorrectly with the probability of each image. [file 13104_2021_5902_MOESM2_ESM.pdf]

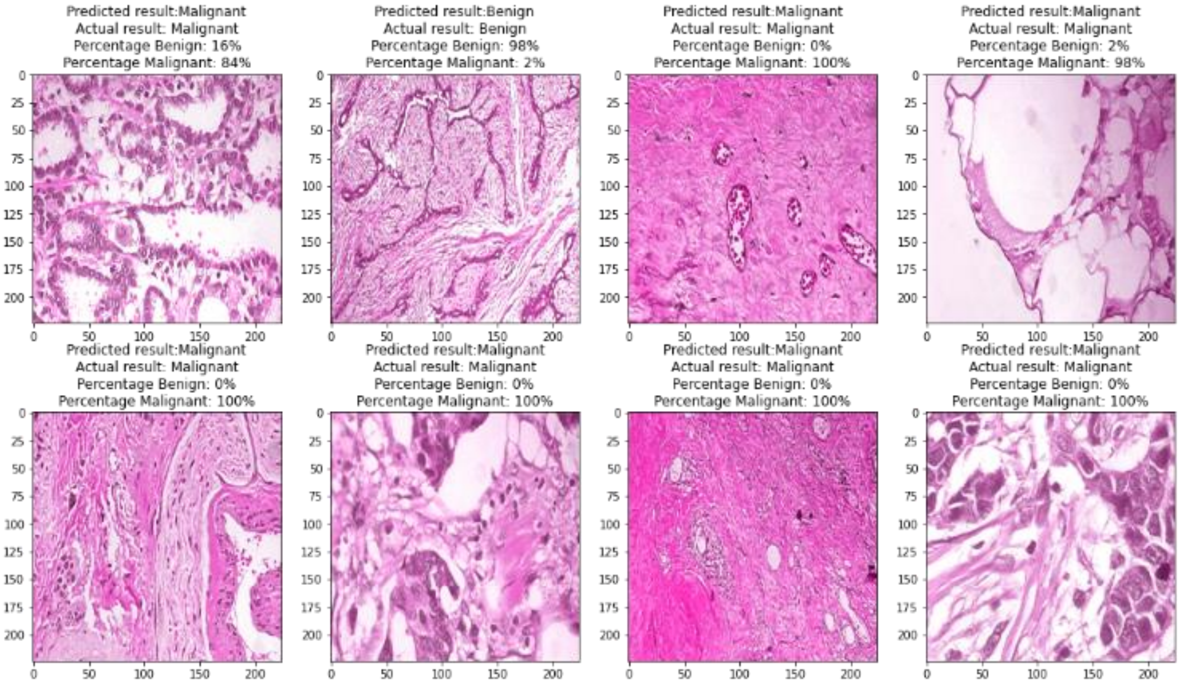

Figure 1 Examples of the images that have been predicted as true-positive or true-negative by the Suggested model

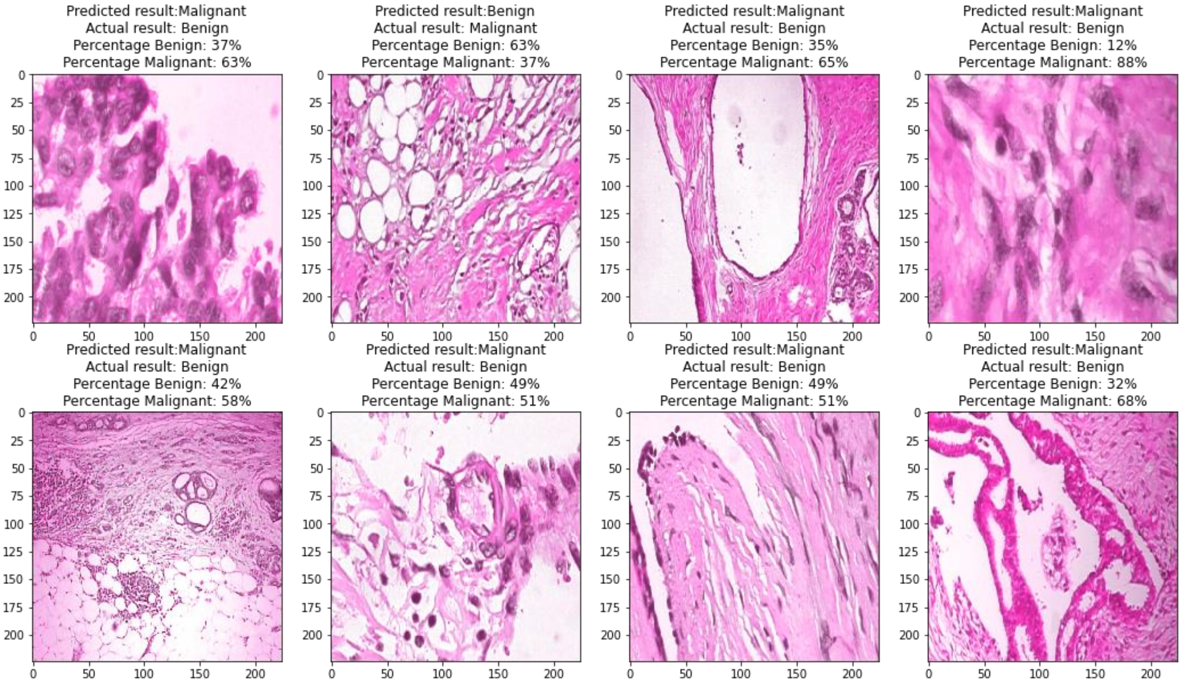

Figure 2 Examples of the images that have been predicted as false-positive or false-negative by the Suggested model
